# Genetic and morphometric differentiation between two morphs of *Haematobosca sanguinolenta* (Diptera: Muscidae) from Thailand

**DOI:** 10.1016/j.crpvbd.2024.100186

**Published:** 2024-06-04

**Authors:** Tanasak Changbunjong, Thekhawet Weluwanarak, Sedthapong Laojun, Gerard Duvallet, Tanawat Chaiphongpachara

**Affiliations:** a Department of Pre-Clinic and Applied Animal Science, Faculty of Veterinary Science, Mahidol University, Nakhon Pathom, 73170, Thailand; b The Monitoring and Surveillance Center for Zoonotic Diseases in Wildlife and Exotic Animals (MoZWE), Faculty of Veterinary Science, Mahidol University, Nakhon Pathom, 73170, Thailand; c Department of Public Health and Health Promotion, College of Allied Health Sciences, Suan Sunandha Rajabhat University, Samut Songkhram, 75000, Thailand; d Center d’Ecologie Fonctionnelle et Evolutive (CEFE), Université Montpellier, Center National de la Recherche Scientifique (CNRS), Ecole Pratique des Hautes Etudes (EPHE), Institut de Recherche pour le Développement (IRD), Université Paul Valéry Montpellier 3, 34199, Montpellier, France

**Keywords:** Geometric morphometrics, Genetic divergence, *Haematobosca sanguinolenta*, Species delimitation, Stomoxyinae

## Abstract

*Haematobosca* is a genus of biting fly within the subfamily Stomoxyinae of the family Muscidae. It is currently recognized to include 16 species worldwide. These species, acting as ectoparasites, are considered to have significant importance in the veterinary and medical fields. To address the color polymorphism related to the genus *Haematobosca* in Thailand, herein, we focused on the normal (legs mainly black) and yellow (legs mainly yellow) morphs of *Haematobosca sanguinolenta* and examined them for genetic differences using three molecular markers: the cytochrome *c* oxidase subunit 1 (*cox*1) and cytochrome *b* (*cytb*) genes from the mitochondrial genome as well as the internal transcribed spacer 2 (ITS2) region from the nuclear ribosomal DNA. In addition, we analyzed wing differences between the two morphs using geometric morphometrics (GM). The genetic divergences between the two morphs showed that *cytb* gene showed the greatest divergence, for which the average distance was 5.6%. This was followed by the combination of *cox*1-*cytb*-ITS2, exhibiting an average divergence of 4.5%, ITS2 with a divergence of 4.1%, and finally *cox*1, showing the lowest divergence of 3.5%. Phylogenetic analyses distinctly separated the two morphs of *H. sanguinolenta*; this separation was supported by high bootstrap values (97–100%). These results were further corroborated by three species delimitation methods, i.e. assemble species by automatic partitioning (ASAP), automated barcode gap discovery (ABGD), and Poisson tree processes (PTP), all of which suggested that the two morphs likely represent separate species. In addition, a GM study identified a statistically significant difference in wing shape between the two morphs of *H. sanguinolenta* (*P* < 0.05). This combination of genetic and morphometric results strongly supports the existence of two distinct species within *H. sanguinolenta* in Thailand.

## Highlights

• Analysis of *cox*1, *cytb* and ITS2 loci revealed genetic differences between two morphs of *Haematobosca sanguinolenta.*
• Wing geometric morphometrics also revealed differences between the two morphs of *H. sanguinolenta.*
• Genetic and morphometric data suggest the existence of two distinct species within *H. sanguinolenta.*

## Graphical abstract

**Figure:**
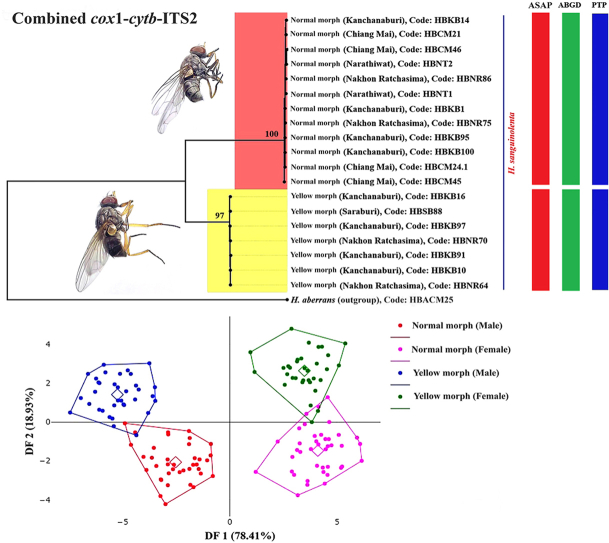

## 1 Introduction

*Haematobosca* Bezzi, 1907 is a genus of biting flies belonging to the subfamily Stomoxyinae within the family Muscidae. As listed by Braack and Pont (2012) and Pont et al. (2020), worldwide, there are currently 16 recognized species of this genus, including *Haematobosca aberrans* Pont, Duvallet & Changbunjong, 2020, *Haematobosca alcis* (Snow, 1891), *Haematobosca atripalpis* (Bezzi, 1895), *Haematobosca angustifrons* (Malloch, 1932), *Haematobosca aurata* Pont & Mihok, 2000, *Haematobosca croceicornis* Pont & Dsouli, 2009, *Haematobosca hirtifrons* (Malloch, 1932), *Haematobosca kangwagyei* (Zumpt, 1967), *Haematobosca latifrons* (Malloch, 1932), *Haematobosca praedatrix* (Enderlein, 1928), *Haematobosca sanguinolenta* (Austen, 1909), *Haematobosca squalida* (Grünberg, 1913), *Haematobosca stimulans* (Meigen, 1824), *Haematobosca uniseriata* (Malloch, 1932), *Haematobosca wooffi* (Zumpt, 1969), and *Haematobosca zuluensis* (Zumpt, 1950). These species are ectoparasites; they feed on the blood of mammals and are considered to be of significant veterinary importance. Their bloodsucking activities pose health risks to both domestic animals and wildlife, including the transmission of pathogens. It was suspected that *H. sanguinolenta* was the vector of the Apicomplexan parasite *Theileria cervi* in Thailand (Changbunjong et al., 2016b). Furthermore, some species can also attack humans (Zumpt, 1973).

Thailand is renowned as being a tropical country rich in biological diversity, supported by a vast array of ecosystems (Pomoim et al., 2022). Despite this biodiversity, detailed knowledge about certain species, including the biting fly genus *Haematobosca*, is still limited. Historically, only one species of *Haematobosca*, *H. sanguinolenta*, was known to exist in Thailand (Zumpt, 1973; Tumrasvin and Shinonaga, 1978; Changbunjong et al., 2012). Nevertheless, recent research has broadened this perspective, and a new species named *H. aberrans* was identified in Chiang Mai Province in the north of Thailand in 2020 (Pont et al., 2020). This species is distinguished by a unique feature, i.e. the absence of the anterior katepisternal seta, which differentiates it from other species in the genus (Pont et al., 2020). Consequently, the number of species of *Haematobosca* documented in Thailand has now increased to two.

Despite the discovery of this second species, the taxonomy of *H. sanguinolenta* in Thailand remains complicated. Zumpt (1973) wrote that this fly is a variable species: the wings can be hyaline or have a brownish tinge, and the legs can range in color from predominantly black to entirely yellow. Tumrasvin and Shinonaga (1978) noted that some *H. sanguinolenta* specimens from Khao Yai National Park in Nakhon Ratchasima Province exhibited unique morphological traits, such as distinctly yellow legs, which set them apart from specimens in other regions of Thailand. However, the presence of the yellow-legged form of *H. sanguinolenta* is not limited to Nakhon Ratchasima; a further study has since revealed its presence across multiple regions of Thailand (Changbunjong et al., 2016c; Ardkhongharn et al., 2023). This underscores the need for further research to clarify the color variation of the species within the genus *Haematobosca*, focusing on the differentiation between two morphs of *H. sanguinolenta*’s legs: one mainly yellow in color and the other mainly black.

Based on investigations of the genetic differences between species, recent analyses have revealed numerous new species of insect vectors such as black flies (Srisuka et al., 2023) and mosquitoes (Phanitchakun et al., 2019). However, the accurate identification of new species through genetic analysis requires the examination of multiple molecular markers to ensure precise differentiation. This necessity arises because a single molecular marker may not adequately distinguish between true speciation and mere regional variations or genetic forms within a species. Geometric morphometrics (GM) provides a valuable complementary technique by revealing unique differences in shape between species. This technique has facilitated the discovery of new species of various insects, such as the ghost spider (Wilson et al., 2021) and caddisfly (Vilarino et al., 2024).

To address the color polymorphism of the genus *Haematobosca* in Thailand, this study focused on the two known morphs of *H. sanguinolenta*, examining them for genetic differences using three molecular markers: the cytochrome *c* oxidase subunit 1 (*cox*1) and cytochrome *b* (*cytb*) genes from the mitochondrial genome as well as the internal transcribed spacer 2 (ITS2) region from the nuclear ribosomal DNA. In addition, GM was employed to analyze the differences between the wings of the two morphs of *H. sanguinolenta*. The results of this research are crucial for determining whether the observed variations in *H. sanguinolenta* represent mere morphological differences or indicate the presence of distinct species. These insights are also invaluable to the surveillance of insect vectors, which are of significant veterinary and medical importance in Thailand.

## 2 Materials and methods

### 2.1 Collection and identification of *H. sanguinolenta*

**Fig. 1:**
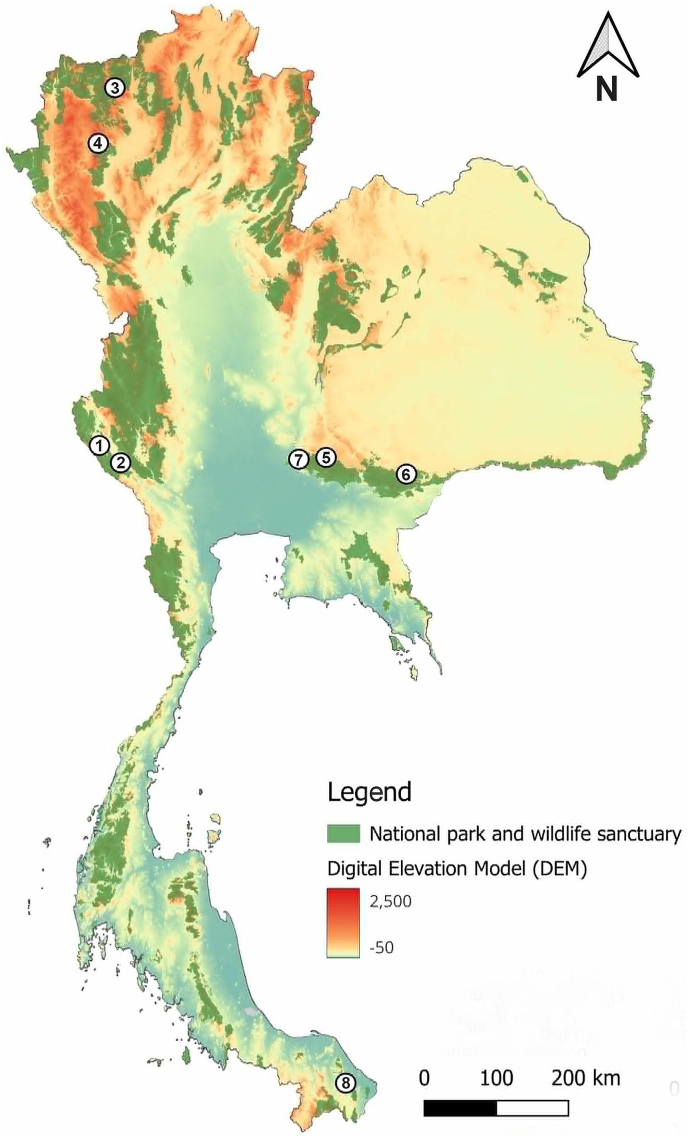
Map showing collection sites in five provinces across five geographical regions of Thailand: the Western region, including Thong Pha Phum (1) and Sai Yok (2) districts in Kanchanaburi Province; the Northern region, including Chiang Dao (3) and Mae Wang (4) districts in Chiang Mai Province; the Northeastern region, including Pak Chong (5) and Soeng Sang (6) districts in Nakhon Ratchasima Province; the Central region, including Kaeng Khoi District (7) in Saraburi Province; and the Southern region, including Rangae District (8) in Narathiwat Province.

Specimens of *H. sanguinolenta* were collected from five geographical regions across Thailand using five Nzi traps (Mihok, 2002) in 2023 (Fig. 1). The geographical coordinates of the collection sites are provided in Table 1. At each site, the traps were strategically placed near hosts such as cattle or buffaloes, thus ensuring optimal placement for fly capture. The traps were set during daylight hours, from 6:00 h to 18:00 h, for a period of two days. Subsequently, the collected flies were euthanized by freezing at −10 °C; they were then individually stored in 1.5 ml microcentrifuge tubes. The specimens were transported to the Vector-Borne Diseases Research Unit at the Faculty of Veterinary Science, Mahidol University, and stored at −20 °C upon arrival at the laboratory, prior to morphological identification.

**Table 1:** Details of collection sites and numbers of the normal and yellow morphs of *H. sanguinolenta* used for the genetic and geometric morphometric (GM) analyses.

Collection site			Date	No. of normal morph used				No. of yellow morph used			
Province	District	Coordinates		Genetic analysis		GM analysis		Genetic analysis		GM analysis	
				♂	♀	♂	♀	♂	♀	♂	♀
Kanchanaburi	Thong Pha Phum	14°39′30″N, 98°32′20″E	Nov 2023	1	1	1	2	1	1	4	3
	Sai Yok	14°25′52″N, 98°48′35″E	Nov 2023	1	1	15	15	1	1	15	14
Chiang Mai	Chiang Dao	19°22′30″N, 98°44′09″E	Nov 2023	2	3	10	10	1	1	8	3
	Mae Wang	18°38′28″N, 98°31′26″E	Nov 2023	0	0	4	5	0	0	1	0
Nakhon Ratchasima	Pak Chong	14°30′05″N, 101°26′28″E	Jul 2023	0	1	6	6	0	2	5	11
	Soeng Sang	14°16′54″N, 102°28′16″E	Jun 2023	1	0	2	2	0	0	0	0
Saraburi	Kaeng Khoi	14°28′39″N, 101°05′33″E	Jun 2023	0	0	0	0	1	1	2	2
Narathiwat	Rangae	06°15′14″N, 101°41′34″E	Jun 2023	2	0	0	0	0	0	0	0
Total				7	6	38	40	4	6	35	33

**Fig. 2:**
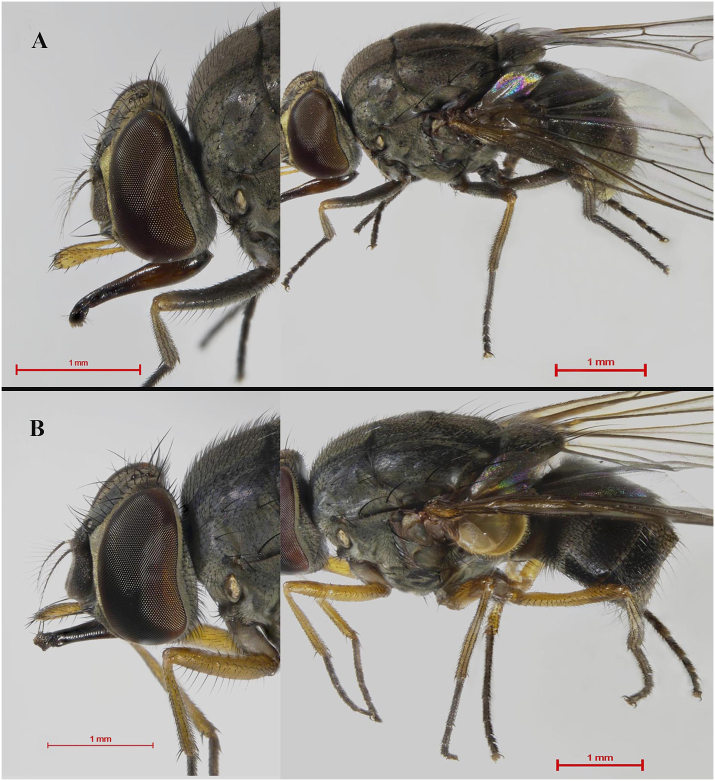
The two distinct morphs of *H. sanguinolenta*: the “normal morph,” characterized by mainly black legs (**A**), and the “yellow morph,” characterized by mainly yellow legs (**B**).

The specimens were examined under a stereomicroscope (Nikon AZ 100, Nikon Corporation, Tokyo, Japan). The taxonomic keys of medically important flies in Thailand (Tumrasvin and Shinonaga, 1978) and stomoxyine biting flies of the world (Zumpt, 1973) were used for identification. After identification, the specimens were categorized into two distinct morphs based on leg color: the “normal morph,” distinguished by mainly black legs (Fig. 2A), and the “yellow morph,” characterized by mainly yellow legs (Fig. 2B).

### 2.2 Specimen preparation

*Haematobosca sanguinolenta* individuals of both identified morphs were randomly chosen for genetic and morphometric analyses (Table 1). For the genetic analysis, two to three legs from each specimen were detached from the thorax. The legs were preserved in 90% ethanol contained in 1.5-ml microcentrifuge tubes and subsequently stored at −20 °C. For the morphometric analysis of the wings, the left-hand wings were dissected, carefully mounted on microscope slides using Hoyer’s medium, and photographed using a digital camera connected to the stereomicroscope. A scale-bar was included in each wing image to facilitate accurate measurement.

### 2.3 DNA extraction, amplification, and sequencing

DNA was extracted from the legs of individual specimens using the DNeasy® Blood & Tissue Kit (Qiagen, Hilden, Germany), following the manufacturer’s instructions. Three molecular markers were used for molecular investigation: the *cox*1 and *cytb* genes from the mitochondrial genome, and the ITS2 region from the nuclear ribosomal DNA. Approximately 658 bp of the *cox*1 gene was amplified using a primer pair consisting of LepF1 (5′-ATT CAA CCA ATC ATA AAG ATA TTG G-3′) and LepR1 (5′-TAA ACT TCT GGA TGT CCA AAA AAT CA-3′) (Hebert et al., 2004). The *cytb* samples, 592 bp in length, were amplified using a primer pair consisting of CB-J10933 (5′-GTT TTA CCT TGA GGA CAA ATA TC-3′) and CB-N11526 (5′-TTC AAC TGG TCG AGC TCC AAT TCA-3′) (Simon et al., 1994). The ITS2 samples, which ranged from 432 to 437 bp in length, were amplified using a primer pair consisting of ITS2A (5′-TGT GAA CTG CAG GAC ACA T-3′) and ITS2-F (5′-TAT GCT TAA ATT CAG GGG GT-3′) (Simon et al., 1994). The amplification reactions were performed in a volume of 25 μl, containing 5 μl of genomic DNA (approximately 50–100 ng), 1 × PCR buffer, 1.5 mM MgCl_2_, 1.5 units of Platinum Taq DNA Polymerase (Invitrogen), 0.1 μM of each forward and reverse primer, and 0.2 mM of dNTPs; the remainder of the mixture consisted of distilled water. The thermal cycling conditions for *cox*1 included an initial denaturation at 94 °C for 1 min, followed by 5 cycles at 94 °C for 30 s, 40 s at 45 °C, and 1 min at 72 °C. This was followed by 35 cycles at 94 °C for 30 s, 40 s at 55 °C, and 1 min at 72 °C, with a final extension step at 72 °C for 10 min followed by an indefinite hold at 4 °C. The PCR reactions for *cytb* and ITS2 started with denaturation at 94 °C for 4 min, followed by 35 cycles at 94 °C for 40 s, 40 s at 58 °C (*cytb*) or 61 °C (ITS2), and 1 min at 72 °C, with a final extension step at 72 °C for 10 min followed by an indefinite hold at 4 °C. Each set of PCR reactions included a negative control without DNA and positive controls containing DNA from *Stomoxys calcitrans*. The PCR products were subjected to electrophoresis, visualized on a 1.5% agarose gel stained with Midori Green DNA stain (Nippon Gene, Tokyo, Japan) and imaged under an ImageQuant LAS 500 imager (GE Healthcare Japan Corporation, Tokyo, Japan). The detection of a band at the anticipated amplicon size was considered a positive result. Subsequently, the PCR products were sent to the Solgent Company in Daejeon, South Korea for purification and DNA sequencing. Specimens that failed to amplify for any molecular marker were excluded from that marker’s analysis.

### 2.4 Molecular analysis

The obtained chromatograms were initially processed using BioEdit software version 7.2.5 (Hall, 1999) to produce a consensus sequence for each specimen. These consensus sequences were then manually aligned using the ClustalW software (Larkin et al., 2007) and further edited using MEGA software version 11 (Tamura et al., 2021) to prepare them for subsequent analysis. To assess the genetic divergences within and between the two morphs of *H. sanguinolenta*, the Kimura 2-parameter (K2P) distance model (Kimura, 1980) was calculated within MEGA. Phylogenetic trees were constructed using the maximum likelihood (ML) method based on the best nucleotide substitution model for each gene, thus giving visual representations of these relationships. The reliability of the phylogenetic tree branches was evaluated through bootstrap analysis with 1000 replicates, which ensured that there was robust support for the inferred genetic relationships. For the phylogenetic analysis of *cox*1 (658 bp), *cytb* (592 bp), and ITS2 (437 bp) (Table 2), a sequence for *Musca domestica* (Diptera: Muscidae: Muscinae) retrieved from GenBank was used as the outgroup, and *H. aberrans* (Diptera: Muscidae: Stomoxyinae) from our Mae Wang, Chiang Mai collection was used as being representative of other *Haematobosca* species groups in Thailand. For the phylogenetic analysis of the combined *cox*1-*cytb*-ITS2 dataset, *H. aberrans* was used as the outgroup due to the absence of the combined sequences for other fly species in the GenBank database. Moreover, neighbor-joining (NJ) and Bayesian phylogenetic trees were constructed using the combined *cox*1-*cytb-*ITS2 dataset to confirm the genetic relationship between the two morphs of fly samples. The NJ tree underwent bootstrap analysis with 1000 replicates using MEGA software version 11. In parallel, the Bayesian tree was generated with MrBayes software version 3.2.7 (Ronquist et al., 2012), employing a general time reversible (GTR) model with a gamma distribution. The Bayesian analysis was executed over 40,000 generations, with a sampling frequency of 100 generations.

**Table 2:** GenBank accession numbers for *Haematobosca* species in Thailand and the outgroup used for construction of the phylogenetic trees.

Species	*cox*1			*cytb*			ITS2		
	*n*	GenBank ID	Source	*n*	GenBank ID	Source	*n*	GenBank ID	Source
*H. sanguinolenta* normal morph	13	PP359534–PP359546	This study	12	PP378154–PP378165	This study	13	PP359423–PP359435	This study
*H. sanguinolenta* yellow morph	10	PP359547–PP359556	This study	10	PP378166–PP378175	This study	9	PP359436–PP359444	This study
*H. aberrans*	3	PP359557–PP359559	This study	2	PP378176, PP378177	This study	1	PP359445	This study
*Musca domestica* (outgroup)	1	OK065531	GenBank	1	KU932174	GenBank	1	EF560189	GenBank

Three species delimitation methods were used: assemble species by automatic partitioning (ASAP) (Puillandre et al., 2021); automated barcode gap discovery (ABGD) (Puillandre et al., 2012); and the single rate Poisson tree processes (PTP) method (Kapli et al., 2017). Species delimitation is the process employed to differentiate between populations of the same species and to identify distinct species within a group of organisms. The ASAP method, a distance-based approach, was implemented using its dedicated web server (https://bioinfo.mnhn.fr/abi/public/asap/). This method effectively distinguishes between interspecific and intraspecific variations, thus facilitating the identification of putative molecular operational taxonomic units. ABGD analysis partitions DNA sequence datasets into clusters of similar taxa, establishing a range of maximum intraspecific divergence values (P) without any presupposition of species hypotheses. This analysis was performed using the default parameter values (distance based on the JC69 model (Jukes and Cantor, 1969); Pmin = 0.001, Pmax = 0.1, steps = 10, X = 1.5, Nb bins = 20) on its web server (https://bioinfo.mnhn.fr/abi/public/abgd/abgdweb.html). In contrast, the PTP method, which is tree-based or “phylogeny-aware,” infers putative species boundaries from a given phylogenetic input tree. This analysis, too, was conducted on a dedicated web server (https://mptp.h-its.org/#/tree). Furthermore, genetic differentiation estimates (*F*_*ST*_), based on the combined *cox*1-*cytb*-ITS2 dataset for comparisons between the two morphs, were derived by calculating conventional *F*-statistics from haplotype frequencies using Arlequin version 3.5 (Excoffier and Lischer, 2010). Upon completion of the molecular analysis, all of the sequences used in this study were deposited in GenBank; the accession numbers are provided in Table 2.

### 2.5 Wing geometric morphometrics

**Fig. 3:**
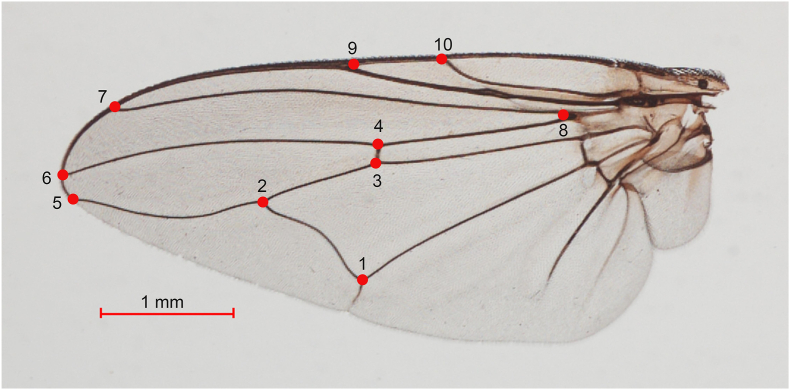
Locations of the 10 landmarks on the left-hand wing of *H. sanguinolenta* used in the geometric morphometric (GM) analysis.

The GM analysis of the wings was conducted using XYOM (XY Online Morphometrics) software (Dujardin and Dujardin, 2019) on 148 specimens of *H. sanguinolenta*. These specimens consisted of 78 specimens of the normal morph (38 males and 40 females) and 70 specimens of the yellow morph (35 males and 35 females). The wing images for both morphs were transformed from morphological to morphometric data by digitizing 10 landmarks located at the intersections of the wing veins, as shown in Fig. 3. The choice of these landmark locations drew on previous research (Changbunjong et al., 2016a; Ardkhongharn et al., 2023) that had demonstrated their effectiveness in classifying blood-feeding flies. To assess the precision of the landmark digitization (also called repeatability), 10 images of each morph of *H. sanguinolenta* were randomly selected and redigitized by the same person. The repeatability of the measurements was quantified using the Procrustes ANOVA method (Klingenberg and McIntyre, 1998), which provided a statistical estimate of the shape repeatability.

Wing size was estimated based on the centroid size (CS), which was derived from the square root of the sum of the squared distances between the centroid and each landmark (Bookstein, 1991). Differences in wing size between the two morphs of *H. sanguinolenta* were analyzed using non-parametric ANOVA (1000 replicates) with Bonferroni correction; the significance threshold was set at *P* = 0.05, with values less than this being considered significant.

In order to obtain the wing shape variables for use in discriminant analysis (DA), Procrustes superimposition was performed using generalized Procrustes analysis. The DA was then used to produce factor maps that visually represented the variations in wing shape between the two morphs of *H. sanguinolenta*. Following the DA, Mahalanobis distances were calculated as shape distance metrics to quantify the differences between the two morphs. The statistical significance of the wing shape differences, as determined from these distances, was evaluated using a non-parametric permutation test with 1000 permutations and Bonferroni correction for multiple comparisons; a significance threshold of *P* = 0.05 was again set. In addition, cross-validation classification was conducted to verify the accuracy of the classification of the specimens into their respective morphs. This involved sequentially excluding each specimen from the analysis and then assigning it to the most likely group based on the Mahalanobis distance.

## 3 Results

### 3.1 Molecular analysis

#### 3.1.1 Genetic divergences

In our molecular analysis of *H. sanguinolenta*, we identified genetic divergence both within and between the normal and yellow morphs of the species, as detailed in Table 3. Target *H. sanguinolenta* sequences included *cox*1 (*n* = 23; normal morph, *n* = 13, yellow morph, *n* = 10), *cytb* (*n* = 22; normal morph, *n* = 12, yellow morph, *n* = 10), ITS2 (*n* = 22; normal morph, *n* = 13, yellow morph, *n* = 9), and the combination *cox*1-*cytb*-ITS2 (*n* = 19; normal morph, *n* = 12, yellow morph, *n* = 7).

**Table 3:** Intra- and intermorph genetic divergences for the *cox*1, *cytb*, ITS2 and combined *cox*1-*cytb*-ITS2 sequences for the normal and yellow morphs of *H. sanguinolenta*, and *H. aberrans.*

*Haematobosca* spp.	Genetic divergence (average percentage)			
	*cox*1	*cytb*	ITS2	Combined *cox*1-*cytb*-ITS2
(i) Intramorph genetic divergence				
*H. sanguinolenta* normal morph	0–0.9 (0.3)	0–0.7 (0.3)	0–0 (0.0)	0–0.5 (0.2)
*H. sanguinolenta* yellow morph	0–0.5 (0.1)	0–0.7 (0.4)	0–0.2 (0.1)	0–0.4 (0.2)
*H. aberrans*	0–0.9 (0.6)	0.2–0.2 (0.2)	NC	NC
(ii) Intermoph genetic divergence				
Normal *vs* yellow morph of *H. sanguinolenta*	3.3–4.1 (3.5)	5.4–6.0 (5.6)	3.9–4.2 (4.1)	4.3–4.7 (4.5)
*H. sanguinolenta* normal morph *vs H. aberrans*	9.7–10.8 (10.2)	8.8–9.6 (9.1)	6.9–6.9 (6.9)	8.9–9.1 (8.9)
*H. sanguinolenta* yellow morph *vs H. aberrans*	9.6–10.2 (9.8)	8.0–8.6 (8.3)	9.9–9.9 (9.9)	9.1–9.2 (9.2)

*Note*: “NC” stands for “not calculated.” This refers to the cases for which only one sequence was available, making comparisons within groups impossible.

Concerning intramorph genetic divergence, the *cox*1 sequences exhibited an average divergence of 0.3% (range 0–0.9%) for the normal morph and 0.1% (range 0–0.5%) for the yellow morph. The *cytb* sequences had an average divergence of 0.3% (range 0–0.7%) for the normal morph and 0.4% (range 0–0.7%) for the yellow morph. The ITS2 sequences revealed no divergence (0%) for the normal morph and a low average divergence of 0.1% (range 0–0.2%) for the yellow morph. From analysis of the combined *cox*1-*cytb*-ITS2 sequences, we found an average divergence of 0.2% for both morphs. In contrast, the intermorph genetic divergences between the two morphs were greatest for the *cytb* sequences, for which the average value was 5.6% (range 5.4–6.0%). This was followed by the combined *cox*1-*cytb*-ITS2 sequences, which showed an average divergence of 4.5% (range 4.3–4.7%) and the ITS2 sequences at 4.1% (range 3.9–4.2%); the *cox*1 sequences exhibited the lowest divergence at 3.5% (range 3.3–4.1%).

Table 3 also includes the intraspecific genetic distances for *H. aberrans* and the intermorph genetic distances between the two morphs of *H. sanguinolenta* and *H. aberrans*.

#### 3.1.2 Phylogenetic trees

**Fig. 4:**
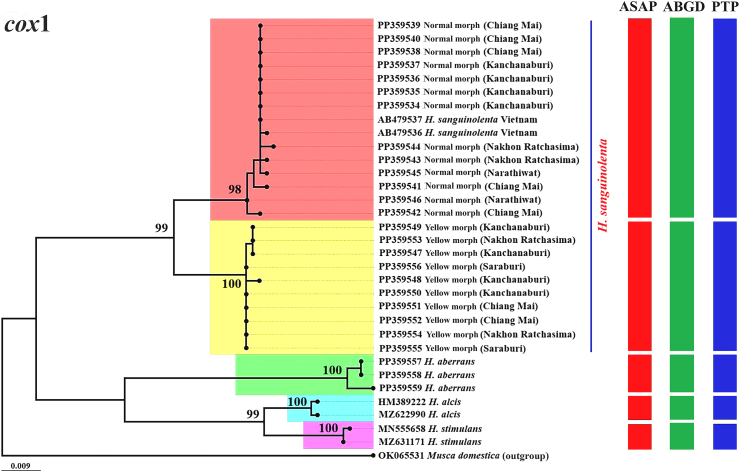
Maximum likelihood phylogenetic tree based on the *cox*1 gene sequences from both the normal and yellow morphs of *H. sanguinolenta* and *H. aberrans* in Thailand (using sequences sourced from this study) together with *H. alcis* and *H. stimulans* from other countries (using sequences sourced from GenBank). *Musca domestica* (OK065531) is used as the outgroup. Bootstrap values exceeding 90% are displayed on the branches. Species delineations are demarcated with vertical bars according to three distinct methods: the ASAP method (red bars), the ABGD method (green bars), and the PTP method (blue bars). The model of nucleotide substitution employed for constructing this tree was the General Time Reversible (GTR) model with gamma distribution and invariant sites (GTR+G+I).

**Fig. 5:**
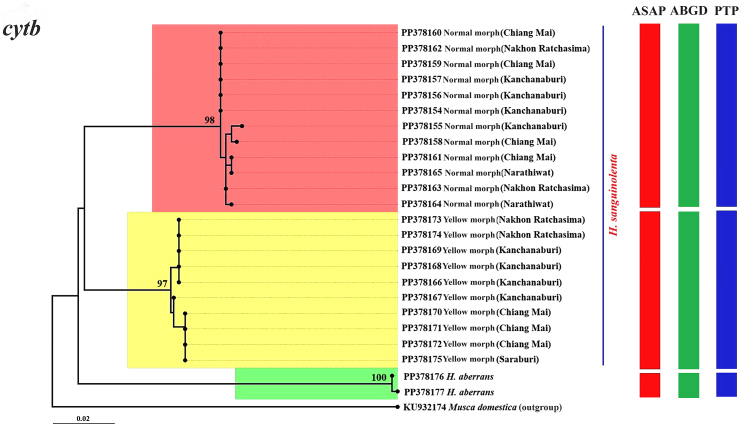
Maximum likelihood phylogenetic tree based on the *cytb* gene sequences from both the normal and yellow morphs of *H. sanguinolenta* and *H. aberrans* in Thailand (using sequences sourced from this study). *Musca domestica* (KU932174) is used as the outgroup. Bootstrap values exceeding 90% are displayed on the branches. Species delineations are demarcated with vertical bars according to three distinct methods: the ASAP method (red bars), the ABGD method (green bars), and the PTP method (blue bars). The model of nucleotide substitution employed for constructing this tree was the Tamura-Nei model with gamma distribution (TN93+G).

**Fig. 6:**
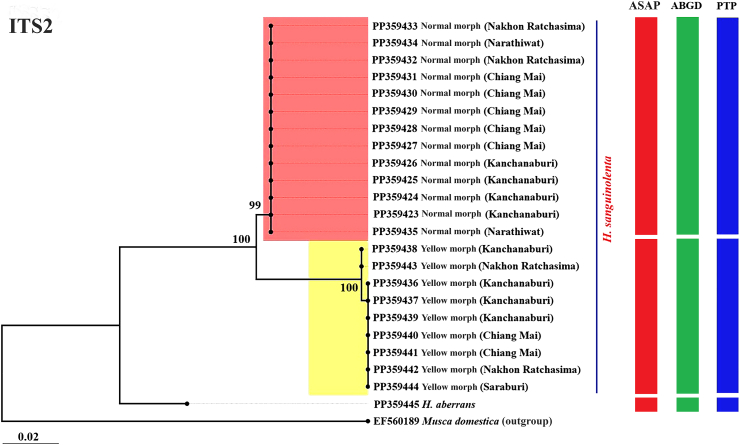
Maximum likelihood phylogenetic tree based on the ITS2 sequences from both the normal and yellow morphs of *H. sanguinolenta* and *H. aberrans* in Thailand (using sequences sourced from this study). *Musca domestica* (EF560189) was used as the outgroup. Bootstrap values exceeding 90% are displayed on the branches. Species delineations are demarcated with vertical bars according to three distinct methods: the ASAP method (red bars), the ABGD method (green bars), and the PTP method (blue bars). The model of nucleotide substitution employed for constructing this tree was the Tamura model (T92).

**Fig. 7:**
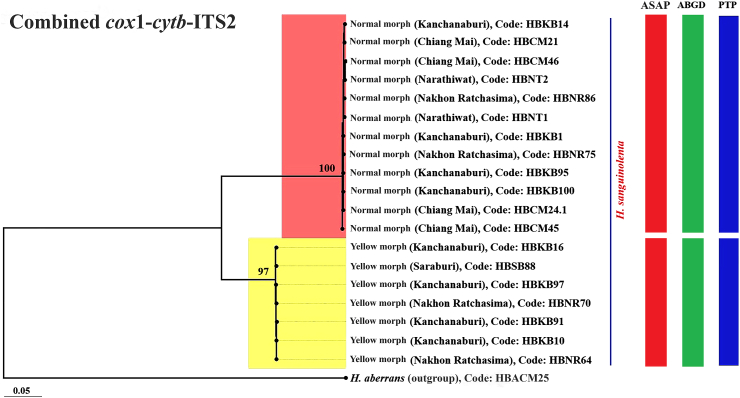
Maximum likelihood phylogenetic tree based on the combined *cox*1-*cytb*-ITS2 sequences from both the normal and yellow morphs of *H. sanguinolenta* (using sequences generated in this study). A sequence for *H. aberrans* in Thailand (also generated in this study) was used as the outgroup. Bootstrap values exceeding 90% are displayed on the branches. Species delineations are demarcated with vertical bars according to three distinct methods: the ASAP method (red bars), the ABGD method (green bars), and the PTP method (blue bars). The model of nucleotide substitution employed for constructing this tree was the General Time Reversible (GTR) model with gamma distribution (GTR+G).

The ML phylogenetic trees constructed from the *cox*1, *cytb*, and ITS2 and the combined *cox*1-*cytb*-ITS2 sequences clearly separated the two morphs of *H. sanguinolenta*; this result was supported by high bootstrap values of 97–100% (Fig. 4, Fig. 5, Fig. 6, Fig. 7). The trees derived from the *cox*1 sequences illustrated a clear genetic difference between these morphs of *H. sanguinolenta* and other *Haematobosca* species, including *H. aberrans* in Thailand, as well as *H. alcis* and *H. stimulans*, which are reported in other regions. This is in line with the findings from the analyses based on *cytb* and ITS2 sequences, which also clearly distinguished the two *H. sanguinolenta* morphs from *H. aberrans*, placing them in distinct clades. Similar patterns are observed in both NJ and Bayesian phylogenetic trees based on the combined dataset, as shown in Supplementary Figs. S1 and S2.

#### 3.1.3 Species delimitation

Species delimitation was conducted using the three methods ASAP, ABGD, and PTP, to determine boundaries between species units. The results of the specimen sequence partitioning using these methods, organized according to the normal and yellow morphs of *H. sanguinolenta*, are presented alongside the phylogenetic trees in Fig. 4, Fig. 5, Fig. 6, Fig. 7.

**Fig. 8:**
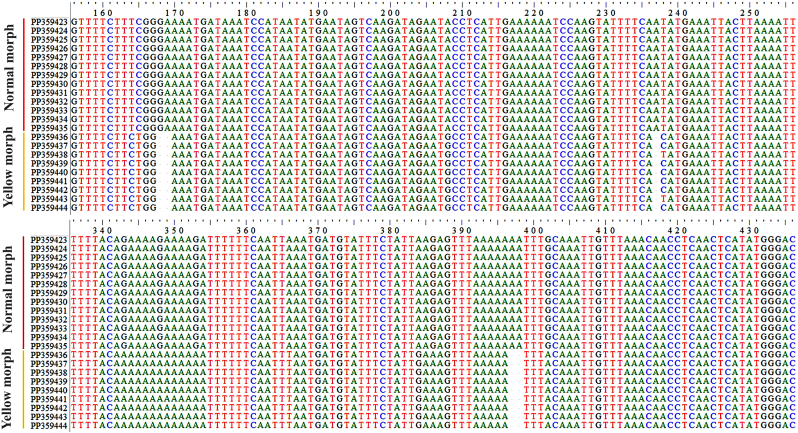
Positions of the insertion-deletion (indel) events in the ITS2 region sequences that distinguish between the normal and yellow morphs of *H. sanguinolenta*. The indel events were identified at nucleotide positions 168, 169, 237, 397, and 398.

In the ASAP analysis, a lower ASAP score suggests a more accurate partitioning. This analysis was based on simple distances (p-distances) and was able to differentiate between the normal and yellow morphs of *H. sanguinolenta* using all of the different genetic markers tested. The ASAP scores for *cox*1, *cytb*, and ITS2 were 2.00, 1.50, and 1.50, respectively; the combined *cox*1-*cytb*-ITS2 sequences had an ASAP score of 1.00. In addition, an insertion-deletion (indel) event was identified in the ITS2 region, further distinguishing the two morphs from one another (Fig. 8).

Using the ABGD method, the specimen sequences were classified into two groups corresponding to the normal and yellow morphs of *H. sanguinolenta*, with the observed prior maximal distance (P) values were 0.002 for *cox*1, 0.005 for *cytb*, 0.003 for ITS2, and 0.002 for the combined sequences. The PTP method also delineated two operational taxonomic units aligned with the normal and yellow morphs of *H. sanguinolenta*. The best scores indicating single coalescent rates were noted for *cox*1 (97.37), *cytb* (59.86), ITS2 (15.20), and the combined sequences (110.74).

The examination of genetic differentiation revealed statistically significant differences between the two morphs (7.10% of variation, *F*_*ST*_ = 0.071, *P* = 0.041, based on 10,000 permutations).

### 3.2 Wing geometric morphometric analysis

The analysis of two sets of wing measurements, which were taken from the same wing images by the same person, demonstrated a high level of consistency between the digitized landmarks, with a repeatability score of 98% and a measurement error of only 2%. This suggests that the digitization of these landmarks was performed with a high degree of precision.

#### 3.2.1 Wing size analysis

**Fig. 9:**
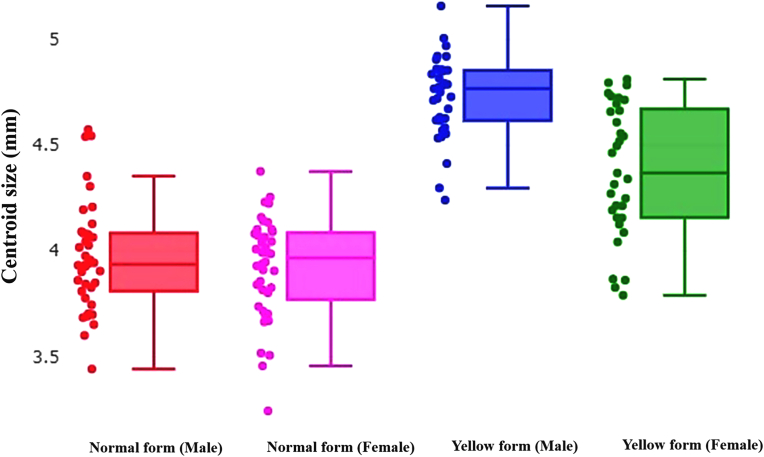
Quantile boxes of wing centroid size (CS) variations in male and female *H. sanguinolenta* of both the normal and yellow morphs. The horizontal line in each box represents the median and separates the 25th from the 75th quartile.

The quantile box plot analysis depicted in Fig. 9 illustrates variations in the wing CS of both male and female *H. sanguinolenta* of both the normal and yellow morphs. The wing CS of the yellow morph of *H. sanguinolenta* (male = 4.73 mm, female = 4.38 mm) was found to be larger than that of the normal morph (male = 3.97 mm, female = 3.92 mm), as shown in Table 4. Statistically significant differences (*P* < 0.05) were observed in the wing CS between the two morphs of *H. sanguinolenta*. In addition, significant distinctions (*P* < 0.05) were found between males and females of the yellow morph of *H. sanguinolenta*.

**Table 4:** Comparison of wing centroid size (CS) between the normal and yellow morphs of *H. sanguinolenta* together with statistical details for each morph.

*H. sanguinolenta*	Sex	*n*	Mean (mm)	Variance	SD
Normal morph	Male	38	3.97^A^	0.07	0.26
	Female	40	3.92^A^	0.06	0.24
Yellow morph	Male	35	4.73^B^	0.04	0.19
	Female	33	4.38^C^	0.10	0.31

*Note*: Values that are statistically significantly different (*P* < 0.05) are denoted by different superscript capital letters. *Abbreviation*: SD, standard deviation.

#### 3.2.2 Wing shape analysis

**Fig. 10:**
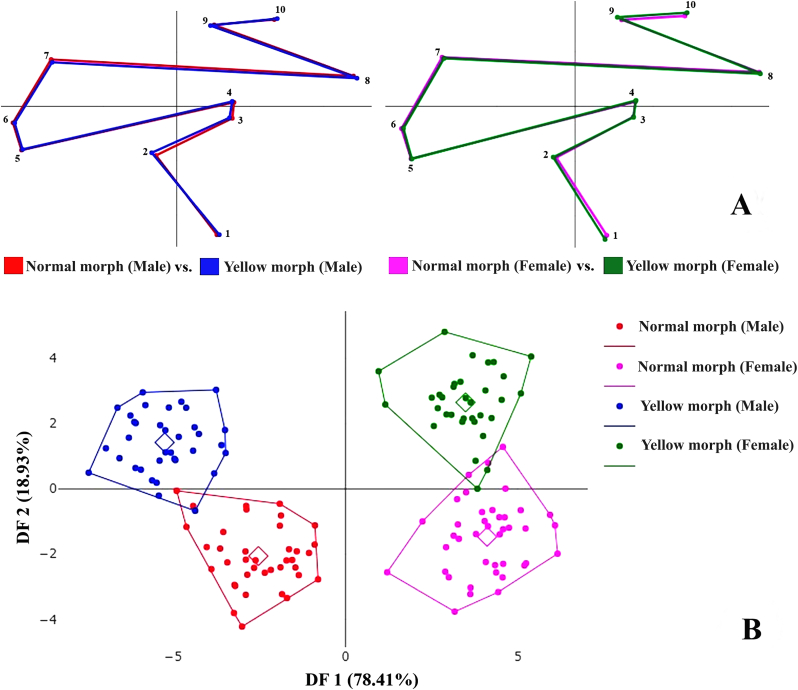
**A** Superposition of the aligned mean anatomical landmark positions for male and female *H. sanguinolenta* of both the normal and yellow morphs. **B** Factor maps based on discriminant analysis of wing shape variations between normal and yellow morphs of *H. sanguinolenta*. The points within each polygon mark individual wing samples, with the small squares indicating the mean group positions. The horizontal axis corresponds to the first discriminant factor (DF1), and the vertical axis corresponds to the second discriminant factor (DF2).

After conducting a generalized Procrustes analysis, graphical representations of the wing shapes were created for both males and females of both the normal and yellow morphs of *H. sanguinolenta* through the superimposition of aligned mean configurations. These graphical representations of the wing showed that the most visible displacements distinguishing between the normal and yellow morphs of *H. sanguinolenta* occur at landmarks 2 and 3 for males and at landmarks 1 and 2 for females (Fig. 10A). Furthermore, factor maps derived from DA depicting variations in wing shape between the normal and yellow morphs of both male and female *H. sanguinolenta* were produced; these are illustrated in Fig. 10B. The first two discriminant factors accounted for a substantial portion of the total variance (87.34%) with DF1 contributing 78.41% and DF2 contributing 18.93%. An analysis of wing shape differences between the normal and yellow morphs of both sexes, based on pairwise comparisons of Mahalanobis distances, indicated significant differences across all groups, as detailed in Table 5. A validated classification test based on Mahalanobis distances revealed that wing shape could be used to separate the two morphs of *H. sanguinolenta* with a high degree of accuracy: the overall accuracy was 94.52% (range 92.50–97.14%; Table 6).

**Table 5:** Mahalanobis distances between the normal and yellow morphs of *H. sanguinolenta*.

	Normal morph (♂)	Normal morph (♀)	Yellow morph (♂)	Yellow morph (♀)
Normal morph (♂)	–			
Normal morph (♀)	6.84	–		
Yellow morph (♂)	4.67	9.79	–	
Yellow morph (♀)	7.64	4.39	8.91	–

*Note*: All pairs showed significant differences (*P* < 0.05).

**Table 6:** Cross-validated classification of the normal and yellow morphs of *H. sanguinolenta* based on wing shape.

Morph	Sex	Percentage correct assignation (assigned/observed)
Normal morph	Male	94.74 (36/38)
	Female	92.50 (37/40)
Yellow morph	Male	97.14 (34/35)
	Female	93.94 (31/33)
Overall performance		94.52 (138/146)

## 4 Discussion

This study was conducted to resolve the color variation concerning the biting fly *H. sanguinolenta* in Thailand. Two distinct morphs of *H. sanguinolenta* were identified; these were co-occurring in several areas of Thailand, including Thong Pha Phum and Sai Yok districts in Kanchanaburi Province, Chiang Dao and Mae Wang districts in Chiang Mai Province, and Pak Chong District in Nakhon Ratchasima Province. Notably, all these study sites were situated within or close to forested areas (see Fig. 1), suggesting a shared forest habitat for the flies. The coexistence of these morphs across diverse geographical regions raises intriguing questions about the ecological factors driving their distribution. One hypothesis could be the influence of microhabitat preferences within forested environments, where specific features may favor one morph over the other. Additionally, the presence of host preferences within these forested areas may also play a role in shaping the distribution patterns of these morphs. In the present study, we observed a higher occurrence of the yellow morph in the Pak Chong district of Nakhon Ratchasima Province compared to other areas. Interestingly, Pak Chong is inhabited by diverse wild animals, including sambar deer, whereas other regions are primarily populated by domestic animals such as cattle and buffaloes. Further exploration into the ecological dynamics of the forest habitats, including factors such as microclimate variations and host availability, could provide valuable insights into the mechanisms underlying the observed color variation in *H. sanguinolenta* populations. Despite their coexistence, our genetic analysis using the ITS2 sequences showed no signs of hybridization, as indicated by the minimal variation observed among sequences.

The presence of the two morphs raises the possibility that these morphs may define two different species within *H. sanguinolenta*. For instance, the recent identification of a new fly species in Thailand named *H. aberrans* was based on morphological characteristics, notably the absence of the anterior katepisternal setae (Pont et al., 2020). While distinct leg coloration in *H. sanguinolenta* populations is noticeable, this difference concerns only coloration. However, the difference in leg color has served as a taxonomic key for the identification of other *Haematobosca* species such as *H. stimulans*, which has black legs, and *H. kangwagyei*, which has completely yellow legs (Zumpt, 1973). Comprehensive genetic investigations are crucial to definitively determine whether these morphs represent distinct species or are simply variations in morphology.

The investigation into intermorph genetic divergence based on *cox*1 sequences revealed a 3.5% genetic difference between the normal and yellow morphs of *H. sanguinolenta*. This difference exceeds the 3% threshold commonly used for species delimitation based on the *cox*1 gene, as proposed by Hebert et al. (2003). However, Zhang et al. (2017) noted that significant genetic variation in the *cox*1 gene, often exceeding 3%, can be observed with comprehensive sampling, which can impact the outcome of species delimitation. This highlights the necessity of analyzing multiple genes or DNA regions for accurate species identification. Further investigations into the genetic distances of the *cytb* and ITS2 loci, as well as an analysis of combined *cox*1-*cytb*-ITS2 sequences, demonstrated differences of 5.6%, 4.1%, and 4.5%, respectively, between the normal and yellow morphs. Notably, the *cytb* gene displayed the greatest genetic difference. The *cytb* gene codes a peripheral protein located on the inner mitochondrial membrane of eukaryotic cells, that is involved in electron transport within the mitochondrial respiratory chain. This gene has been reported to contain valuable information regarding the evolution of various animal species, including insects. This makes it a valuable molecular marker that can aid in the classification of species (Seddigh and Darabi, 2018). In China, for example, the *cytb* gene has been effectively used as a marker for identifying sand fly species (Chen et al., 2023).

The detection of an insertion-deletion (indel) event within the ITS2 region provides crucial evidence for there being a distinction between the two morphs of *H. sanguinolenta*. This event is significant because deletions in the ITS2 region can influence the maturation of ribosomal RNA subunits (Good et al., 1997). Moreover, ITS2 exhibits low sequence variation within species but considerable variation between species, making it an excellent marker for phylogenetic studies across a broad range of insect taxa. These findings, supported by the phylogenetic analyses of the *cox*1, *cytb*, and ITS2 sequences and the combination of these sequences, clearly distinguish between the two morphs of *H. sanguinolenta*, suggesting that there are two distinct species previously grouped under *H. sanguinolenta* in Thailand. No spatial genetic patterns were observed in either the normal or yellow morphs, indicating that geographical distribution does not influence the genetic differentiation between these morphs. Thus, the observed genetic differences were attributed to distinct morphological forms. This conclusion is supported by the results obtained using three species delimitation methods, ASAP, ABGD, and PTP, which all indicated that the two morphs likely represent separate species. These results are in line with the findings from a previous study on *Lutzia chiangmaiensis* in Thailand, which was identified as a new mosquito species due to morphological and, subsequently, genetic differences (Phanitchakun et al., 2019). Moreover, the genetic differentiation index (*F*_*ST*_) between the two morphs, based on the combined *cox*1-*cytb*-ITS2 dataset, suggested significant genetic differentiation, underscoring the distinct genetic characteristics of the morphs that might be evolutionarily meaningful (Liu et al., 2019).

The GM methods used for the wing analysis supported the results of our genetic investigations and again revealed significant differences between the normal and yellow morphs of *H. sanguinolenta*. Although the analysis of wing size showed that the yellow morph has significantly larger wings than the normal morph, it is crucial to recognize that wing size differences might not solely indicate distinct species characteristics. These variations can be attributed to a range of environmental factors including temperature, larval density, and the quality and quantity of food, which influence the developmental stages of insects (Phanitchat et al., 2019); for example, Baleba et al. (2019) found that *S. calcitrans* developed larger wings under conditions of lower larval density and good substrate quality.

Wing shape is recognized as a unique characteristic of each flying insect species and is influenced by each species’ distinct genetics. Previous studies have demonstrated that various blood-feeding flies exhibit distinct wing shapes that are identifiable using the GM method; these species include *Stomoxys* flies such as *S. bengalensis*, *S. calcitrans*, and *S. sitiens* (Changbunjong et al., 2023), as well as horse flies (*Tabanus* spp.) such as *T. megalops*, *T. rubidus*, and *T. striatus* (Changbunjong et al., 2021). Our findings indicate that the normal and yellow morphs of *H. sanguinolenta* display significant differences in wing shapes across both males and females. The high accuracy (94.52%) of our validated classification test in distinguishing these morphs based on wing shape suggests that these differences are not merely morphological variations but do, indeed, represent distinct species.

## 5 Conclusion

Genetic analysis of the *cox*1 and *cytb* genes from the mitochondrial genome of *H. sanguinolenta*, together with analysis of the ITS2 region from the nuclear ribosomal DNA, revealed distinct differences between the normal and yellow morphs of this species. These results were supported by an analysis of wing shape based on wing GMs. Together, this evidence strongly suggests the existence of two distinct species within *H. sanguinolenta* in Thailand. The yellow morph can be distinguished from the traditionally recognized *H. sanguinolenta*, which is characterized by mainly black legs. We intend to further investigate the morphological and biological characteristics of the yellow morph of *H. sanguinolenta* in Thailand.

## Funding

This research was funded by Specific League Funds (SLF-2566) from 10.13039/501100020874Mahidol University.

## Ethical approval

The collection of *H. sanguinolenta* and all related experimental procedures in this study were reviewed and approved by the Animal Care and Use Committee of the Faculty of Veterinary Science, Mahidol University (Ref. MUVS-2021-11-50).

## CRediT authorship contribution statement

**Tanasak Changbunjong:** Conceptualization, Methodology, Formal analysis, Investigation, Writing – original draft, Writing – review & editing, Visualization, Supervision. **Thekhawet Weluwanarak:** Conceptualization, Methodology, Formal analysis, Investigation, Writing – original draft, Writing – review & editing, Visualization, Supervision. **Sedthapong Laojun:** Conceptualization, Methodology, Formal analysis, Investigation, Writing – original draft, Writing – review & editing, Visualization. **Gerard Duvallet:** Conceptualization, Writing – original draft, Writing – review & editing, Visualization. **Tanawat Chaiphongpachara:** Conceptualization, Methodology, Formal analysis, Investigation, Writing – original draft, Writing – review & editing, Visualization, Supervision.

## Declaration of competing interests

The authors declare that they have no known competing financial interests or personal relationships that could have appeared to influence the work reported in this paper.

## Data Availability

The data supporting the conclusions of this article are included within the article. The newly generated sequences were submitted to the GenBank database under the accession numbers PP359534-PP359559 (*cox*1 gene), PP378154-PP378177 (*cytb* gene), and PP359423-PP359445 (ITS2 region).
