# Supplementary material for: Genetic and morphometric differentiation between two morphs of Haematobosca sanguinolenta (Diptera: Muscidae) from Thailand
Source: Curr Res Parasitol Vector Borne Dis. 2024 Jun 4;6:100186. doi: 10.1016/j.crpvbd.2024.100186 (PMC11252610; doi:10.1016/j.crpvbd.2024.100186)

Supplementary Figure S1. Neighbor-joining (NJ) tree based on the combined *cox*1-*cytb*-ITS2 sequences from both the normal and yellow morphs of *H. sanguinolenta* (using sequences generated in this study). Sequence from *H. aberrans* in Thailand (also sourced from this study) was used as the outgroup. Bootstrap values > 90% are displayed on the branches. Species delineations are demarcated with vertical bars according to three distinct methods: the ASAP method (red bars), the ABGD method (green bars), and the PTP method (blue bars).

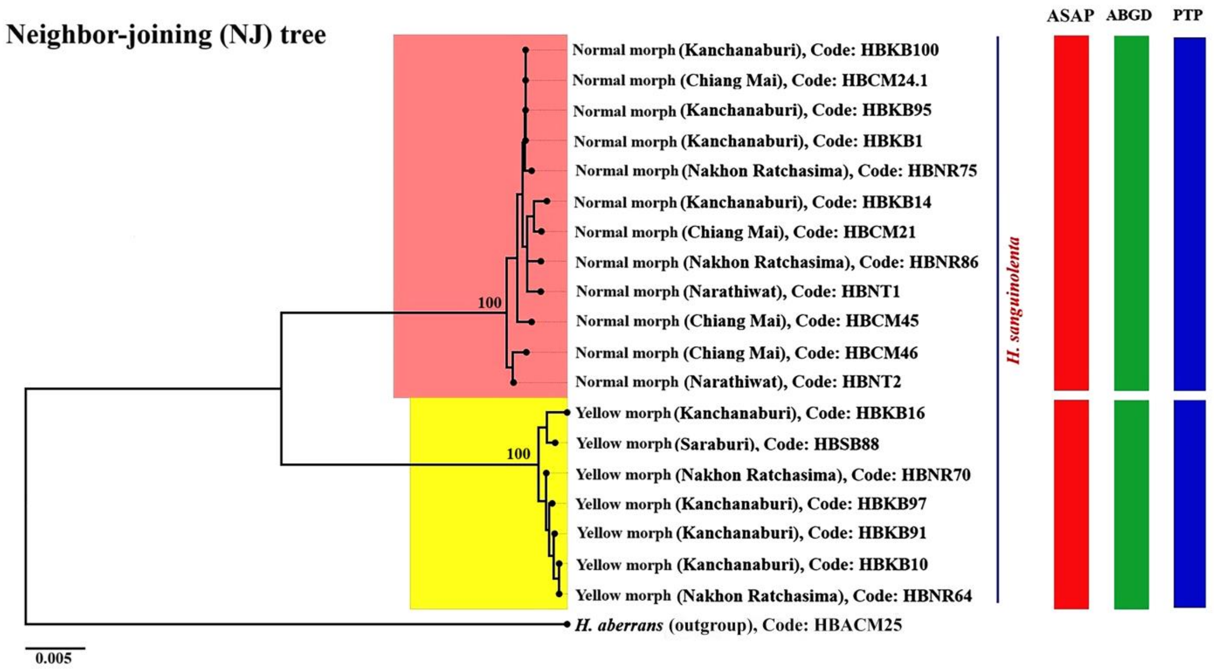

Supplementary Figure S2. Bayesian tree based on the combined *cox*1-*cytb*-ITS2 sequences from both the normal and yellow morphs of *H. sanguinolenta* (using sequences generated in this study). Sequence from *H. aberrans* in Thailand (also generated in this study) was used as the outgroup. Posterior probability values exceeding 0.9 are displayed on the branches. Species delineations are demarcated with vertical bars according to three distinct methods: the ASAP method (red bars), the ABGD method (green bars), and the PTP method (blue bars).

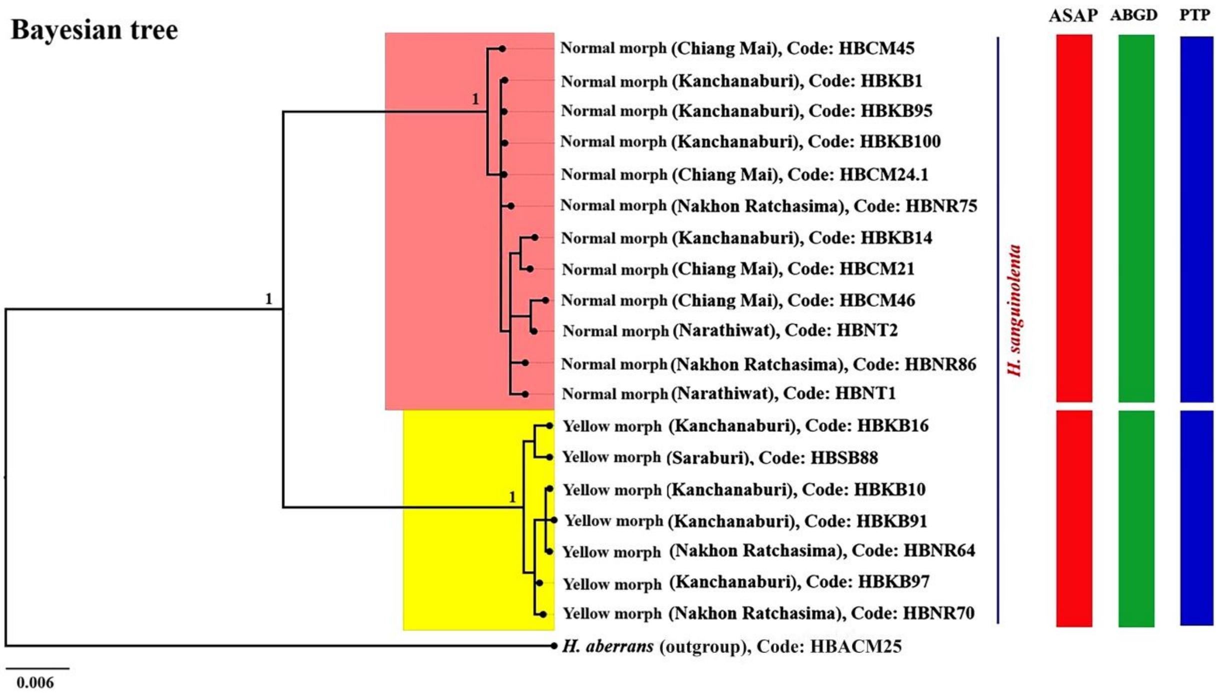

Supplement: Multimedia component 1 [file mmc1.pdf]
